# Exploring the Influence of Ethical Leadership on Voice Behavior: How Leader-Member Exchange, Psychological Safety and Psychological Empowerment Influence Employees’ Willingness to Speak Out

**DOI:** 10.3389/fpsyg.2018.01718

**Published:** 2018-09-12

**Authors:** Yixin Hu, Liping Zhu, Mengmeng Zhou, Jie Li, Phil Maguire, Haichao Sun, Dawei Wang

**Affiliations:** ^1^School of Psychology, Shandong Normal University, Jinan, China; ^2^School of Management, Shanghai University, Shanghai, China; ^3^Department of Computer Science, National University of Ireland, Maynooth, Ireland

**Keywords:** ethical leadership, leader-member exchange, psychological safety, psychological empowerment, voice behavior

## Abstract

The study of voice behavior examines the inclination of staff and team members to speak up and contribute ideas to the team. In this article, we investigate how factors such as leader-member exchange (LMX), psychological safety and psychological empowerment influence such behavior. Our findings, which are based on a sample of 308 employees working for a state-owned telecommunications company in China, indicate that ethical leadership promotes employees’ voice behavior through enhanced LMX, which also leads to greater feelings of psychological safety and psychological empowerment. The theoretical and practical implications of these results are discussed.

## Introduction

In China, enterprises are increasingly shifting the focus of their reforms toward stimulating the development of organizational effectiveness under the guidance of the long-term planning system. It is anticipated that innovative ideas and constructive suggestions originating internally will inject new vitality into their ongoing development, allowing them to better face new global opportunities and challenges. Accordingly, greater attention is being paid to issues such as employee empowerment, on which endogenous innovation depends. Research has shown positive associations between individual psychological empowerment, innovative behavior and organizational performance (Spreitzer, 1995; Spreitzer et al., 1999; Sun et al., 2012).

Employee voice behavior has been shown to play an important role in determining the competitive advantage and success of an organization (Locke et al., 1997; Duan, 2011; Morrison, 2011; Maynes and Podsakoff, 2014), leading to growing interest in this topic over the past 20 years (LePine and Van Dyne, 1998; Premeaux and Bedeian, 2003; Ng and Feldman, 2012; Chen and Hou, 2016). This line of research has scored some notable practical successes, for example, enhancing airline safety statistics by encouraging airplane crews to speak up in plain language about any perceived dangers (Bienefeld and Grote, 2012).

One of the important factors which has been shown to influence voice behavior in an organizational environment is that of “ethical leadership.” Walumbwa and Schaubroeck (2009), for instance, analyzed the data of 894 employees and 222 direct supervisors, and found that ethical leadership promoted greater voice behavior among followers. Liang (2010) also found a positive effect of ethical leadership on voice behavior. Brown et al. (2005) defined ethical leadership as “the demonstration of normatively appropriate conduct through personal actions and interpersonal relationships, and the promotion of such conduct to followers through two-way communication, reinforcement, and decision-making.” Compared with other leadership styles, such as transactional leadership, transformational leadership and authentic leadership, it has some unique characteristics (Brown et al., 2005). For example, an ethical leader who demonstrates high moral values including integrity, trustworthiness, honesty, is one who acts in a fair and principled manner, and is genuinely concerned for their employees, as well as setting, communicating and reinforcing high ethical standards (Hansen et al., 2013; Lu and Guy, 2014).

Research has shown that ethical leadership exerts a positive impact on employees’ attitudes, so as to help them to create moral identities, and develop citizenship-style behaviors toward their organizations (Brown and Treviño, 2006; Lu et al., 2011; Walumbwa et al., 2011b; Zhu et al., 2015; Chen and Hou, 2016). Leaders who are ethical affect their followers’ attitudes by demonstrating moral behaviors such as honesty, trustworthiness and fairness, and by giving them more opportunities to engage in voice behaviors. A study conducted by Saunders et al. (1992) has shown that the more employees perceive their leaders to be responsive to their voice input, the more likely they are to engage in subsequent voice behavior. Employees who are promoted can then go on to reciprocate these positive behaviors, leading to a virtuous cycle (Walumbwa and Schaubroeck, 2009; Walumbwa et al., 2011a; Liang, 2014).

The question arises as to the precise mechanism by which ethical leadership influences the voice behavior of employees (Walumbwa and Schaubroeck, 2009; Chen and Hou, 2016). It has been suggested that sense of obligation and psychological safety mediate the relationship between ethical leadership and voice behavior (Walumbwa and Schaubroeck, 2009; Liang, 2014). Our present study applies this idea to a Chinese context, and takes it forward. On one hand, we use the concept of leader-member exchange (LMX) to understand voice behavior according to social exchange theory. On the other hand, we use psychological empowerment and psychological safety to understand voice behavior from the perspective of social information processing theory. We also investigate the interaction between these two separate mechanisms.

## Theoretical Background and Hypotheses Development

### Ethical Leadership and Voice Behavior

Employee voice behavior is generally defined as “promotive behavior that emphasizes expression of constructive challenge intended to improve rather than merely criticize” (Van Dyne and LePine, 1998). We use a two-dimensional scale for voice behavior, namely promoting voice behavior and repressing voice behavior (Liang et al., 2012). The recent upsurge of voice behavior research underlies its practical importance in the development of enterprises and government (LePine and Van Dyne, 1998; Burris, 2012; Chen and Hou, 2016). Voice behavior refers to the behavior of employees who challenge the working status and make constructive changes (LePine and Van Dyne, 1998). It is a kind of construction-oriented communication behavior, which aims to improve the working situation. Many researchers have realized the importance of emphasizing the initiative of employees, and voice behavior is one of them (Duan and Zhong, 2005).

Voice behavior has important research significance for both organizations and individuals. For organizations, providing opportunities for employees to contribute advice can lead to a greater perception of fairness (Duan and Zhong, 2005). Research has shown that increased voice behavior is associated with greater sense of control and job satisfaction, though the relationship is not a linear one (Hunton et al., 1998). For individuals, voice behavior is negatively correlated with job stress and positively correlated with job performance (Ng and Feldman, 2012). Other studies have shown that voice behavior can improve employee performance (Song et al., 2017).

Although existing research has shown a link between ethical leadership and voice behavior (e.g., Chen and Hou, 2016), the question remains as to how exactly such leadership exerts its effect. Social learning theory has been used to give an explanation: employees observe their leaders’ behaviors and use it as a reference (Brown et al., 2005; Stouten et al., 2013). As early as 2000 years ago, a prototype concept of ethical leadership and its influence was already mentioned in China. In his famous work “The Analects,” Confucius advised that “If the ruler himself is upright, all will go well even though he does not give orders. But if he himself is not upright, even though he gives orders, they will not be obeyed.” Confucius pointed out that the most effective way to govern was to set a good example, namely the moral model, which can guide subordinates via osmosis.

According to the social learning perspective, the positive effects of ethical leadership are embodied in the following ways: (a) Leaders influence the ethical behavior of followers through modeling. They give guidance and convey uninterrupted high moral standards to employees, as well as deep interaction, thereby establishing a long-term trust and reciprocity relationship which benefits the organization; (b) Ethical leadership is associated with its own positive characteristics (e.g., honesty, trustworthiness, fairness, and care). Leaders encourage their followers to demonstrate moral behaviors and express themselves in the workplace. When employees find themselves in such a friendly and fair environment, they tend to demonstrate more prosocial behaviors, including voice behavior.

### Leader–Member Exchange (LMX)

In the field of voice behavior research, the relationship between superior and subordinate is considered to be a key variable influencing employee voice behavior (Detert and Burris, 2007; Wang et al., 2010). LMX is defined as the quality of exchange between a supervisor and an employee (Graen and Scandura, 1987). The scale of LMX used in this paper is a one-dimensional scale. This level of quality varies along a continuum. For example, leaders may engage in certain high-quality social exchanges that are based on trust, open communication, and compassionate concern, whereas with others, they may engage in lower-quality, functional exchanges (Erdogan et al., 2006). The quality of the relationship can also be impacted by the frequency of the interaction between leaders and followers (Dienesch and Liden, 1986). LMX theory has received substantial attention in the organizational sciences (Nahrgang et al., 2009; Walumbwa et al., 2011c), being used to examine the influence of leaders on employees, and the quality of the relationship between them.

In the present study, we explore the idea that ethical leadership contributes to the formation of high-quality LMX. On one hand, evidence suggests that a leadership style which promotes ethics, altruism, and empowerment is helpful to the formation of high quality LMX relations (Walumbwa et al., 2010). On the other hand, guided by social exchange theory (Blau, 1964), we know that employees tend to develop high-quality relationships with the people with whom they interact; the more frequent the interactions, the stronger the relationship (Dienesch and Liden, 1986). With regard to ethical leadership, leaders who are honest and trustworthy will show care to their employees and be favored by subordinates, leading to a greater frequency of interaction. Thus, ethical leaders are also more concerned with establishing trusting relationships with followers. Employees who perceive more support and greater freedom in taking decisions, and who experience their working environments positively, will have a sense of obligation (Liden and Graen, 1980); these employees are more likely to reciprocate by exhibiting greater voice behavior (Ng and Feldman, 2011). For instance, a study conducted by Stamper et al. (2009) indicates that when individuals develop high-quality LMX with their leaders, they are more likely to perform in pro-organizational ways. Van Dyne et al. (2008) have argued that high quality LMX should further promote voice behavior by employees. Based on these studies, we expect that ethical leaders should enhance LMX with followers, and that such high-quality relationships will promote positive actions such as voice behavior. Our particular interest lies in understanding the psychological mechanisms that underpin this effect.

### Psychological Safety

Psychological safety refers to a shared belief among work unit members that it is safe for them to engage in interpersonal risk taking (Edmondson, 1999). The scale of psychological safety used in this paper is a one-dimensional scale. According to Edmondson, psychological safety in an organization can be affected by both personal factors and institutional factors. Psychological safety goes beyond perceiving and experiencing high levels of interpersonal trust; it also describes a work climate characterized by mutual respect, one in which people are comfortable about expressing their different opinions (Walumbwa and Schaubroeck, 2009).

With regard to voice behavior, this can be viewed as a behavior involving risks. Employees take interpersonal risks into account (LePine and Van Dyne, 1998; Duan, 2011). For instance, they will worry that their voice behavior will lead to a misunderstanding or reprisal by their colleagues and leaders, causing a deterioration in relationships (Dutton et al., 1997; Milliken et al., 2003). Status may be adversely affected.

Psychological safety plays an important role in determining how such risks are evaluated (Detert and Burris, 2007). Specifically, when employees perceive a high level of psychological safety, this will dampen their assessment of negative risks associated with voice behavior. For this reason, psychological safety is regarded as one of the necessary preconditions for employee voice behavior to occur (Ashford et al., 1998; Detert and Burris, 2007; Liang et al., 2012). Previous studies have also suggested that perceptions of psychological safety mediate the relationship between superior-subordinate LMX and voice behavior, as well as mediating the relationship between leadership behavior (ethical leadership and transform leadership) and voice behavior (Detert and Burris, 2007; Van Dyne et al., 2008; Walumbwa and Schaubroeck, 2009). In a review of the definitions, dimensions, and related research on the antecedents and outcomes of psychological safety, Newman et al. (2017) reported that leadership which enhanced employees’ psychological safety, both individually and within teams, led to increased voice behavior (Detert and Burris, 2007; Walumbwa and Schaubroeck, 2009).

According to social information processing theory, people’s behaviors and attitudes are influenced by their social environment (Salancik and Pfeffer, 1978). Employees receive a variety of information in their workplace, which they use to evaluate the risk of voice behavior. Given that ethical leadership is a positive form of leadership, it should lead to the establishment of high quality LMX, causing employees to perceive trust and support from their leaders. This should reduce the perceived level of risk associated with voice behaviors (Treviño et al., 2003). Hofmann and Morgeson (1999) have also suggested that high-quality LMX is typified by openness to the raising of safety concerns. Based on these studies, we propose that ethical leadership can help to eliminate negative concerns relating to personal voice behavior by increasing employees’ perception of psychological safety.

### Psychological Empowerment

Psychological empowerment was defined by Spreitzer (1995) as a form of intrinsic motivation that reflects a proactive orientation and sense of control over work. The scale of psychological empowerment used in this paper is a four dimensional one, comprising meaning (a judgment that one’s work has value), competence (a belief in one’s ability to successfully perform work tasks), self-determination (perceptions that one is free to choose how to carry out work tasks), and impact (a belief in one’s ability to influence organizational outcomes). Psychological empowerment was analyzed as a higher-order factor.

Recent work has focused on the role of psychological empowerment as a mediating variable in various workplace relations (Duan and Zhang, 2012). It has been shown that leadership has a strong effect on psychological empowerment, particularly transformational leadership (Li et al., 2006), and LMX (Liden et al., 2000). Leaders’ supportive behavior and ease of accessibility can significantly improve the level of psychological empowerment. Bae et al. (2011) have argued that if employees think that they have the ability to control their work output and environment, this should promote voice behavior, as well as improving the quality of voice behavior. Other research shows that a trusting and supportive relationship with one’s leader positively impacts the development of psychological empowerment (Seibert et al., 2011; Dulebohn et al., 2012). Building on the above studies, we hypothesize that psychological empowerment acts as a mediating variable between ethical leadership and voice behavior.

As previously discussed, ethical leadership contributes to the formation of high quality LMX relations (Walumbwa et al., 2010). According to social exchange theory, when leaders establish a high quality of relationship with subordinates it can lead them to shift their motivation from short-term oriented individual needs toward long-term oriented collective goals. Furthermore, it has been shown that when employees perceive that they are important to their leaders, it influences their own cognition of their status (Wang et al., 2009; Ni and Chu, 2010; Wang et al., 2010). These studies, conducted in a Chinese context, found that high quality LMX influences employees’ internal identity, stimulating a sense of control and positive expectations for voice behavior. In light of this, we hypothesize that psychological empowerment also mediates the relationship between LMX and voice behavior.

**FIGURE 1:**
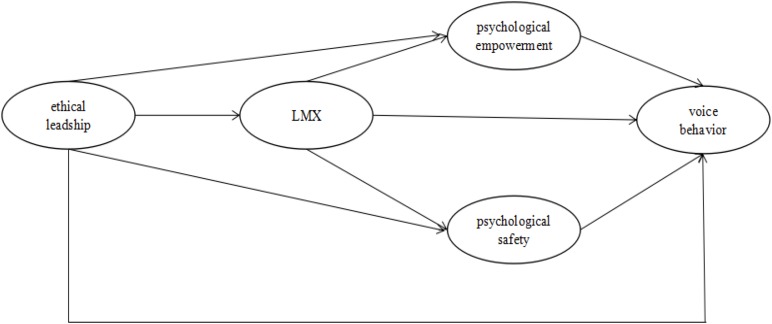
The proposed model of the study.

In the following investigation, we predict that ethical leadership, which we view as positive leadership, will promote the formulation of high quality LMX relationships. Under this condition, employees will have a sense of being an integral member of the organization, thereby enhancing perceptions of psychological safety and psychological empowerment. As a result, they will give more inputs and exhibit positive behaviors toward the development of the organization, including more voice behavior. In order to clarify our proposed mechanism of ethical leadership affecting employees’ voice behavior, we present our research model in **Figure 1**. Based on previous research findings, we put forward the following hypotheses:

Hypothesis 1: Ethical leadership will positively influence voice behavior (1a) and LMX (1b).
Hypothesis 2: Ethical leadership will positively influence psychological empowerment (2a) and psychological safety (2b).
Hypothesis 3: LMX will positively influence psychological empowerment (3a), psychological safety (3b) and voice behavior (3c).
Hypothesis 4: LMX will mediate the relationship between ethical leadership and psychological empowerment (4a), psychological safety (4b), and voice behavior (4c).
Hypothesis 5: Psychological empowerment (5a) and psychological safety (5b) will positively influence voice behavior.
Hypothesis 6: Psychological empowerment (6a) and psychological safety (6b) will mediate the relationship between ethical leadership and voice behavior.
Hypothesis 7: Psychological empowerment (7a) and psychological safety (7b) will mediate the relationship between LMX and voice behavior.

## Materials and Methods

### Sample and Procedure

This study is based on data collected from employees of a state-owned enterprise in the telecommunications industry. To cope with fierce competition, this enterprise was encouraging employees to put forward constructive suggestions that could promote the development of the organization. The questionnaire was conducted in the company’s conference room. Before the start of the questionnaire, we assured participants of full confidentiality. All were informed in writing that their names would not be reported in the data. We then assigned a questionnaire with a unique code to participants. Finally, all completed questionnaires were returned directly to the researcher and assistant.

For the sample recruited from within the telecommunications company’s employees, a total of 320 questionnaires were distributed, with 308 valid questionnaires finally returned, giving a response rate of 96.3%. The population characteristics of the sample were as follows: of the participants who completed the questionnaires, there were 126 males (40.9%) and 182 females (59.1%). Participants ranged in age from 25 years old to 50 years old. In addition, 35.4% had worked more than 10 years in this company, 50.3% had worked there for 4–9 years, and 14.3% for less than 3 years. Of the sample, 97% had completed college or post-high-school education.

### Ethics Statement

The study was carried out in accordance with the ethical standards of the research committee of Shandong Normal University, which is responsible for ethical review within the institution. All participants took part in this research voluntarily, and were given the opportunity to decline to participate. The data obtained from the questionnaire were analyzed anonymously, thus preserving the anonymity of the responses.

### Measures

Although all the measures were originally developed in English, they were later translated into Chinese and applied in the Chinese context. All of the items except Ethical leadership were scored on a 5-point scale ranging from 1 (Strongly Disagree) to 5 (Strongly Agree).

#### Ethical Leadership

Ethical leadership was measured using the 15-item questionnaire developed and validated by Resick et al. (2006). Respondents used a 7-point Likert scale ranging from 1 (Strongly Disagree) to 7 (Strongly Agree) to evaluate the extent of their agreement with each item. The scale featured four dimensions, namely Integrity, Altruism, Collective Motivation and Encouragement. The Cronbach’s alpha for this scale in the study was 0.984. The reliability of the sub-scale is shown in **Table 2**.

#### Employee Voice

Employee voice was measured using an 11-item questionnaire (e.g., “I develop and make recommendations concerning issues that affect my work”) developed by Liang et al. (2012), and comprising two dimensions. The Cronbach’s alpha for this scale in this study was 0.910. The reliability of the sub-scale is shown in **Table 2**.

#### Leader-Member Exchange (LMX)

Leader-Member Exchange was measured using Scandura and Graen’s (1984) LMX-7 scale, including items such as “How effective is your working relationship with your leader?” The Cronbach’s alpha for this scale in this study was 0.939.

#### Psychological Safety

Employees’ perception of psychological safety was measured using a 4-item questionnaire (e.g., “I’m free to express my ideas”) taken from Li and Yan (2007), which was further developed to situate it in a Chinese context. The Cronbach’s alpha for this scale in this study was 0.735.

#### Psychological Empowerment

Employees indicated their levels of psychological empowerment by completing Spreitzer (1995) 12-item questionnaire (e.g., “I have considerable opportunity for independence and freedom in how I do my job”), comprising four dimensions including three items each. The Cronbach’s alpha for this scale in this study was 0.887. The reliability of the sub-scale is shown in **Table 2**.

### Data Analysis

We analyzed the data using SPSS 21.0, AMOS 7.0 and the bootstrap procedure. Descriptive statistics were used to describe the respondents’ demographic profiles, SEM (Structural Equation Modeling) was used to test the proposed hypotheses, and the bootstrap procedure was used to examine direct and indirect effects.

## Results

### Minimization of Common Method Bias

To address the potential problem of common method bias, we applied process control and statistical analysis. For a start, all the data used in this study came from a common source. Applying the method of process control, we reminded the participants that it was an anonymous questionnaire, and we also included some interference in the items. We also analyzed our data for common method biases using statistical techniques. Pooling all the items of the five constructs into a single factor, and conducting the confirmatory factor analysis (CFA) using AMOS, the outcomes of the one-factor model displayed a poor model fit (GFI = 0.74, TLI = 0.81, RMSEA = 0.12, χ^2/df^ = 5.21, *p* < 0.001). This result supports the conclusion that our study does not have the problem of common method biases.

### Confirmatory Factor Analysis

In order to examine the data, we firstly executed a CFA of the variables. Results of the tests of competing CFA models are shown in **Table 1**. As shown, the outcomes of the hypothesized five-factor measurement model (model-1) display a good model fit (χ^2^ = 372.86, χ^2/df^ = 2.12, RMSEA = 0.06, TLI = 0.95, CFI = 0.96), which is better than the alternative models. These results provide support for the discriminant validity of our measures.

**Table 1:** Comparison of measurement models.

Models	χ^2^	df	χ^2/df^	GFI	TLI	CFI	RMSEA	Confidence interval	SRMR
Five-factor model-1	372.86	176	2.12	0.89	0.95	0.96	0.060	(0.093, 0.099)	0.072
Four-factor model-2	420.34	179	2.35	0.88	0.94	0.95	0.066	(0.096, 0.102)	0.085
Three-factor model-3	615.10	182	3.38	0.82	0.89	0.91	0.088	(0.106, 0.112)	0.098
Two-factor model-4	669.79	184	3.64	0.79	0.88	0.89	0.093	(0.118, 0.124)	0.113
One-factor model-5	958.59	184	5.21	0.74	0.81	0.84	0.117	(0.133, 0.138)	0.144

*Note: One-factor model-5: all items were loaded on one factor; Two-factor model-4: LMX, psychological empowerment, psychological safety, and voice behavior were loaded on one factor; Three-factor model-3: LMX, psychological empowerment, and psychological safety were loaded on one factor; Four-factor Model-2: psychological empowerment and psychological safety were loaded on one factor.*

We also evaluated the convergent validity of the variables by examining the composite reliabilities (CR) and average variance extracted (AVE). As shown in **Table 2**, the outcomes of CR varied from 0.764 to 0.964, ensuring a minimum cutoff at 0.60 (Bagozzi and Yi, 1988), while the estimates for the AVE were all above 0.50 (Fornell and Larcker, 1981), apart from a single dimension at 0.444. These results provide support for convergent validity. In addition, the Cronbach’s α of every dimension was above 0.70, indicating high internal consistency and validity of the constructs (Nunnally, 1978).

**Table 2:** Description of CR and AVE.

	Dimension (items)	Cronbach’s α	CR	AVE
Ethical leadership	EL-1(4)	0.963	0.964	0.870
	EL-2(4)	0.973	0.971	0.894
	EL-3(5)	0.955	0.954	0.807
	EL-4(2)	0.963	0.959	0.921
LMX	LMX(7)	0.939	0.938	0.686
Psychological safety	PS(4)	0.735	0.764	0.444
Psychological empowerment	PE-1(3)	0.882	0.883	0.723
	PE-2(3)	0.854	0.856	0.666
	PE-3(3)	0.812	0.824	0.611
	PE-4(3)	0.899	0.903	0.758
Voice behavior	VB-1(6)	0.871	0.873	0.536
	VB-2(5)	0.903	0.904	0.655

*Note: CR, Composite Reliability; AVE, Average Variance Extracted.*

### Descriptive Statistics

The descriptive statistics, internal consistency reliabilities, and correlations among the study variables are shown in **Table 3**. The results show a significant and positive correlation among all the presumed constructs.

**Table 3:** Means, standard deviations, and correlations.

Variable	M	*SD*	1	2	3	4	5
Ethical leadership	5.70	1.15	(0.98)				
Employee voice	3.56	0.62	0.32^∗∗^	(0.91)			
LMX	4.04	0.70	0.69^∗∗^	0.39^∗∗^	(0.94)		
Psychological safety	3.65	0.67	0.42^∗∗^	0.45^∗∗^	0.55^∗∗^	(0.74)	
Psychological empowerment	3.71	0.56	0.49^∗∗^	0.47^∗∗^	0.44^∗∗^	0.45^∗∗^	(0.89)

*Note: ^∗^p < 0.05, ^∗∗^p < 0.01, ^∗∗∗^p < 0.001.*

### Hypotheses Testing

**FIGURE 2:**
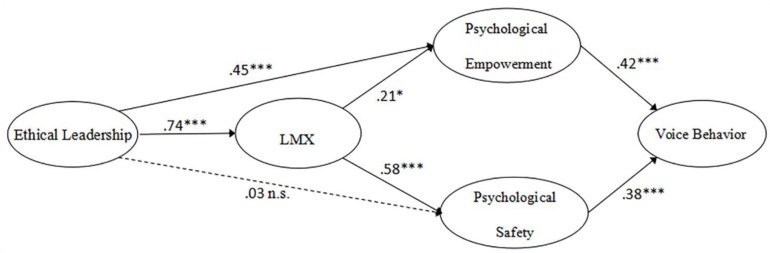
Estimated standardized path coefficients for the research model.

The structural model was derived from the above hypotheses, and the proposed measurement relationships were consistent with the data. The proposed structural model exhibits a good fit to the data: χ^2^= 375.58, *p* < 0.001, CFI = 0.951, IFI = 0.951, TLI = 0.941, RMSEA = 0.067. This result demonstrates that the direct path from LMX to voice behavior is not significant (*β* = 0.04, *p* > 0.05). Accordingly, in the new model we deleted this path. **Figure 2** presents the final structural model and its associated path coefficients.

As indicated in **Figure 2** and **Table 4**, ethical leadership was positively related to LMX [*β* = 0.74, *p* < 0.001, (0.625, 0.797)] and psychological empowerment [*β* = 0.45, *p* < 0.001, (0.211, 0.656)], meaning that hypotheses 1b and 2a were supported. However, ethical leadership didn’t show any significant influence on psychological safety [*β* = 0.03, *p* > 0.05, (−0.121, 0.205)], meaning that hypothesis 2b was not supported. The analyses also reveal that LMX positively influenced psychological empowerment [*β* = 0.21, *p* < 0.05, (0.021, 0.446)] and psychological safety [*β* = 0.58, *p* < 0.001, (0.364, 0.730)], supporting hypotheses 3a and 3b. However, the direct path connecting LMX to voice behavior was not significant [*β* = 0.04, *p* > 0.05, (−0.23, 0.254)], meaning that hypothesis 3c was not supported. In addition, consistent with hypotheses 4a and 4b, the results indicate that LMX mediates the relationship between both ethical leadership and psychological empowerment [*β* = 0.16, *p* < 0.001, (0.015, 0.324)] and between ethical leadership and psychological safety [*β* = 0.43, *p* < 0.001, (0.263, 0.536)]. Hypothesis 4c, however, was not supported (*β* = 0.03, *p* > 0.05). Psychological empowerment [*β* = 0.42, *p* < 0.001, (0.099, 0.647)] and psychological safety [*β* = 0.38, *p* < 0.001, (0.191, 0.599)] positively influenced voice behavior, supporting hypotheses 5a and 5b. Furthermore, the results show that psychological empowerment mediated the relationship between ethical leadership and voice behavior [*β* = 0.19, *p* < 0.001, (0.316, 0.533)], supporting hypothesis 6a, but not hypothesis 6b. They also reveal that psychological empowerment mediated the relationship between LMX and voice behavior [*β* = 0.09, *p* < 0.05, (0.006, 0.286)], and that psychological safety mediated the relationship between LMX and voice behavior [*β* = 0.22, *p* < 0.001, (0.114, 0.419)], supporting hypotheses 7a and 7b. In summary, our findings indicate that ethical leadership influences employees’ voice behavior through LMX and then through psychological mechanisms, such as psychological empowerment and psychological safety.

**Table 4:** Hypotheses test results.

Hypothesized relationship	Proposed model	
	*β*	Test results
H1a Ethical leadership→voice behavior	0.36^∗∗∗^	Supported
H1b Ethical leadership→LMX	0.74^∗∗∗^	Supported
H2a Ethical leadership→psychological empowerment	0.45^∗∗∗^	Supported
H2b Ethical leadership→psychological safety	0.03	Not supported
H3a LMX→psychological empowerment	0.21^∗^	Supported
H3b LMX→psychological safety	0.58^∗∗∗^	Supported
H3c LMX→voice behavior	0.04	Not supported
H4a Ethical leadership→LMX→psychological empowerment	0.16^∗∗∗^	Supported
H4b Ethical leadership→LMX→psychological safety	0.43^∗∗∗^	Supported
H4c Ethical leadership→LMX→voice behavior	0.03	Not supported
H5a Psychological empowerment→voice behavior	0.42^∗∗∗^	Supported
H5b Psychological safety→voice behavior	0.38^∗∗∗^	Supported
H6a Ethical leadership→psychological empowerment→voice behavior	0.19^∗∗∗^	Supported
H6b Ethical leadership→psychological safety→voice behavior	0.01	Not supported
H7a LMX→psychological empowerment→voice behavior	0.09^∗^	Supported
H7b LMX→psychological safety→voice behavior	0.22^∗∗∗^	Supported

*Note: ^∗^p < 0.05, ^∗∗^p < 0.01, ^∗∗∗^p < 0.001.*

## Discussion

On one hand, our results verify that ethical leadership has a highly positive influence on voice behavior, which is consistent with previous studies (e.g., Walumbwa and Schaubroeck, 2009; Liang, 2014). On the other hand, we have also shown that intermediary variables play an important role in this process. Specifically, this kind of positive leadership is conducive to the establishment of high-quality LMX relationships, giving subordinates a sense of psychological safety and psychological empowerment, which in turn promotes voice behavior. The theoretical and practical significance of this study are summarized in the following sections.

### Theoretical Significance

Previous research has investigated the relationship between ethical leadership and voice behavior. For example, Walumbwa and Schaubroeck (2009) found that followers’ perceptions of psychological safety could mediate the relationship between ethical leadership and voice behavior, while Liang (2014) also found that sense of obligation and psychological safety could be used as mediating variables to explain this relationship.

Our findings fit with previous theoretical research efforts and further extend them. Social exchange theory has been used to explain the relationship between leadership and extra-role behavior (Walumbwa et al., 2010), while Detert and Edmondson (2011) pointed out that voice behavior is affected by both the individual and their environment. Accordingly, we used LMX to examine the exchange process between leaders and subordinates from the perspective of social exchange theory. Our results are consistent with those of Van Dyne et al. (2008), supporting the idea that high-quality LMX leads to greater voice behavior. We sought to explain the mediating role of LMX in this relationship based on social exchange theory. We can say that employees value the support of ethical leadership, which leads to a positive emotional connection between leaders and subordinates. This high-quality LMX relationship instills a sense of responsibility in employees to reciprocate, motivating them to come up with new suggestions in the enterprise, and leading them to engage in more voice behaviors.

Voice behavior is not a single process; it has its own challenging characteristics, often accompanied by risk. Promoting greater voice behavior is something that can’t be accomplished overnight. What kinds of conditions can help leaders instill greater voice behavior in their employees? Although the quality of LMX can account for the occurrence of greater “extra role” behavior, it cannot shed light on the finer contextual details of this process.

Focusing on social information processing theory, we selected psychological safety and psychological empowerment as mediators to explore the underlying psychological mechanism of voice behavior. Given that voice behavior may carry risks, psychological safety has been viewed as a precondition for voice behavior (e.g., Ashford et al., 1998; Detert and Burris, 2007; Liang et al., 2012). Our results verified the mediating role of psychological safety and psychological empowerment: high-quality LMX enhances levels of psychological safety and psychological empowerment, which in turn prompts voice behavior. Although ethical leadership promotes the formation of high-quality LMX, we found that the direct path from LMX to voice behavior was not significant. Instead, LMX exerts its impact on voice behavior indirectly, through the experience of psychological safety and psychological empowerment. We speculate that employees’ psychological perception plays an important role in stimulating corresponding voice behavior.

In addition, we further considered the effect of psychological empowerment, finding that it acts as a mediator through two paths. First, ethical leadership has a direct impact on psychological empowerment, which then affects voice behavior. Second, LMX also directly influences psychological empowerment, promoting voice behavior. These observations suggest that more attention should be paid to the role of psychological empowerment in facilitating voice behavior.

In conclusion, our results reveal that the process linking ethical leadership to voice behavior is a complex one, which involves intermediary variables such as LMX, psychological safety and psychological empowerment.

### Practical Significance

With the rapid changes brought about by economic globalization, more and more enterprises are paying attention to the management of employees. By exerting their subjective initiative, employees can inject new vitality into the running of an organization. In particular, voice behavior plays an important role in improving organizational effectiveness. As a result, promoting voice behavior is a significant concern for managers. The results of this study can provide some guidance in the following respects.

On one hand, a greater level of ethical behavior on the part of leaders is highly recommended. Previous studies have shown that leadership style influences employees’ behavior. Our study has offered support for the observation that ethical leadership, which can lead to high quality LMX, actively promotes employees’ voice behavior. Therefore, the more attention managers pay to ethical practice, the better employees will behave. Employees with high quality LMX relationships will change their focus from short-term oriented individual needs toward long-term oriented collective goals. In order to create a positive atmosphere, the management of an enterprise should seek more active forms of leadership, and focus on the building of strong leader-member relationships.

On the other hand, enterprises should also pay more attention to employees’ psychological feelings. In the context of a positive atmosphere, employees can experience higher levels of psychological safety. If they have a sense of security and responsibility toward the enterprise, they will identify personally with the organization and exhibit behavior that is conducive to the development of the enterprise. The feeling of psychological safety, however, is not enough in isolation. In modern society, employees pay great attention to self-growth and personal value. Therefore, organizations should seek to let employees experience greater psychological empowerment, significance of their work, and apply their own initiative.

## Limitations and Avenues for Future Research

Our study has several limitations which also provide some interesting avenues for future research. First, the data in this study were gathered from a single domestic state-owned telecommunications enterprise, which may have different characteristics compared to other enterprises. In order to generalize the conclusions, future research should extend the sample to other enterprises boasting a diverse set of characteristics. In addition, in order to better address the issue of common method bias, future research might collect data at different time points, with an increase in sample size. Second, although our study makes an important contribution by examining how and why ethical leadership is effective in promoting voice behavior, there may be further underlying mechanisms that we have not accounted for. Future studies might cast a broader net in hunting out additional mediating and moderating variables influencing this relationship. Third, our study was mainly focused on the individual level effects of ethical leadership on voice behavior. Future studies could also use a multi-level approach to analyze the effects of ethical leadership, such as, for example, gathering data from work groups as well as from individuals. Fourth, the cross-sectional data used in the current study merely explored the relationship between variables, without addressing the trend of those changes. Future studies might use longitudinal studies to explore possible trends in the interaction between these variables. Finally, there may be important differences between the structure and functioning of Chinese and western organizations. Individualism is given greater priority in western society, whereas interpersonal harmony and collective value orientation are emphasized in traditional Chinese culture. These differences may affect the relationship between ethical leadership and voice behavior. With the number of multinational companies on the increase, the issue of cross-cultural research is an important topic to explore for the future.

## Conclusion

This study explored the ways in which managers’ ethical leadership behavior influences employees’ voice behavior, with particular focus on factors such as LMX, psychological safety and psychological empowerment. The results reveal that ethical leadership promotes employees’ voice behavior both directly and indirectly, namely through enhanced LMX, leading to greater feelings of psychological safety and psychological empowerment. These findings reinforce the value of ethical leadership, and suggest that, in order to enhance long-term returns, organizations should pay more attention to employees’ psychological feelings and personal values.

## Author Contributions

YH designed the study. LZ get the data and wrote the paper. PM and JL revised the paper. MZ and HS collected the data and revised the paper. DW designed the study and wrote the paper.

## Conflict of Interest Statement

The authors declare that the research was conducted in the absence of any commercial or financial relationships that could be construed as a potential conflict of interest.
